# From fiction to science: clinical potentials and regulatory considerations of gene editing

**DOI:** 10.1186/s40169-019-0244-7

**Published:** 2019-10-21

**Authors:** Maria Schacker, Diane Seimetz

**Affiliations:** Biopharma Excellence GmbH, Agnes-Pockels-Bogen 1, 80992 Munich, Germany

**Keywords:** Gene editing, Gene therapy, Drug development, Regulatory strategy, Clinical trials, Artificial intelligence

## Abstract

Gene editing technologies such as CRISPR/Cas9 have emerged as an attractive tool not only for scientific research but also for the development of medicinal products. Their ability to induce precise double strand breaks into DNA enables targeted modifications of the genome including selective knockout of genes, correction of mutations or precise insertion of new genetic material into specific loci. Gene editing-based therapies hold a great potential for the treatment of numerous diseases and the first products are already being tested in clinical trials. The treatment indications include oncological malignancies, HIV, diseases of the hematopoietic system and metabolic disorders. This article reviews ongoing preclinical and clinical studies and discusses how gene editing technologies are altering the gene therapy landscape. In addition, it focusses on the regulatory challenges associated with such therapies and how they can be tackled during the drug development process.

## Background

In recent years, gene editing technologies have been receiving a lot of attention as they emerge as a new treatment modality not only for hereditary genetic conditions but also for a variety of neoplastic diseases. This is largely due to the discovery of the CRISPR/Cas9 (clustered regularly interspaced short palindromic repeat/CRISPR-associated nuclease) system and the recognition of its great potential in both experimental research as well as in the clinical setting [1, 2]. However, gene editing tools are not limited to CRISPR/Cas9, and to date four major types of genome editing technologies are known—meganucleases, zinc finger nucleases (ZFNs), transcription activator-like effector nucleases (TALENs) and CRISPR/Cas9.

Programmable nucleases enable extremely precise genome editing as they can be targeted to a specific location in the genome. At the target site, they are able to generate knockouts or loss of function mutations of selected genes, as well as to correct deleterious mutations by replacing the mutated gene sequence or to knock in transgenes which add a new therapeutic function. The exact mechanisms are beyond the scope of this article and are described in more detail elsewhere [3, 4].

Although meganucleases, ZFNs, TALENs and CRISPR/Cas9 are all technically capable of inducing genetic modifications, the CRISPR/Cas9 system has an important advantage in terms of technical feasibility (Table 1). In the case of meganucleases, ZFNs and TALENs, the nucleases themselves identify and bind to their target site in the genome through protein–DNA interactions [5–7]. Consequently, new proteins have to be engineered for every target site of interest. So far, this is a resource intensive and challenging process, but bioinformatic approaches and artificial intelligence may be able to facilitate the design of these nucleases [8]. In contrast, the Cas9 nuclease used with the CRISPR/Cas9 system is common for all gene editing events, as it is targeted to the DNA via RNA–DNA interactions [1]. In addition to the Cas, a single guide RNA (sgRNA) is required. This sgRNA comprises a scaffold sequence, that binds to the Cas, as well as a ~ 20 nucleotide spacer motif, that binds to the genomic target site [1]. sgRNAs are quick to produce synthetically, greatly facilitating the easy re-targeting of the Cas nuclease to new DNA target sites. Hence, multiplexing gene editing, which is the introduction of more than one DSB in the same cell in only one work step, is also much easier using CRISPR/Cas9, since several distinct sgRNAs can be used with the same Cas nuclease [9]. Although meganucleases, ZFNs and TALENs are also still being utilized as gene editing tools, the simplicity and greater flexibility of the CRISPR/Cas9 system has revolutionized the field. However, it remains to be seen whether the CRISPR/Cas9 system can live up to the high expectations in the clinical setting.

**Table 1:** Summary of characteristics of different gene editing systems

	CRISPR	ZFN	TALEN	Meganuclease
Binding principle	RNA-DNA	Protein–DNA	Protein–DNA	Protein–DNA
Feasibility	Easy	Difficult	Difficult	Difficult
Construction	20 nucleotide sgRNA sequence per target site	Engineering new proteins for every target site	Engineering new proteins for every target site	Engineering new proteins for every target site
Ease of multiplexing	Easy	Difficult	Difficult	Difficult

## Clinical potentials of gene editing

Although gene editing has only recently emerged as a type of gene therapy, gene therapy as such is not a new area of scientific and clinical development. In fact, it has its beginning in as early as the 1970s when scientists first started thinking of using exogenous “good” DNA to replace defective DNA [10]. Almost another 20 years past by, however, until the FDA approved the first gene therapy clinical trial in the US in September 1990. White blood cells of two children suffering from adenosine deaminase deficiency (ADA-SCID) were genetically modified ex vivo to express a functional version of the gene for making adenosine deaminase, and transferred back into the patients. While only one of the children responded to the therapy, this trial certainly laid the foundation for all gene therapies that are already on the market or currently being developed [11].

Fast forward another (almost) 30 years and we are celebrating the results of a number of successful gene therapy clinical trials. Some of the more prominent examples are the two CD19-directed chimeric antigen receptor (CAR) T cell products Kymriah^®^ (Novartis) and Yescarta^®^ (Kite Pharma) indicated for the treatment of patients with relapsed/refractory large B cell lymphoma [12–14], as well as Luxturna^®^, an adeno-associated virus (AAV) vector-based gene therapy indicated for the treatment of biallelic *RPE65* mutation-associated retinal dystrophy [15], and most recently Zolgensma^®^ (Novartis/AveXis) for the treatment of pediatric patients with spinal muscular atrophy caused by a biallelic mutation in the *SMN1* gene [16].

One thing these trials all have in common is, that they are based on traditional gene therapy approaches. These use viral vectors to deliver exogenous DNA that is either expressed transiently or integrates randomly into the genome. Thereby, they bear the risk of insertional mutagenesis which can in theory lead to genome instability and toxicity and eventually cause malignant transformations [17]. In addition, these therapies are restricted to the insertion of new DNA. Using modern gene editing tools, on the other hand, it is now possible to selectively knockout specific genes, correct mutations or insert new genetic material into a specific locus. And even more techniques and tools are being developed that will be described later in this article.

While gene editing systems have originally mainly been used by academic research groups as a tool to study the function and role of genes in a variety of diseases and developmental processes, pharmaceutical companies are now also starting to show an increasing interest in these new technologies and it is likely that the future of gene therapy will be steered by these gene editing tools. Drug development will certainly build on the success of the first gene therapy products, and gene editing offers a much more versatile toolbox than traditional gene therapy.

Altogether, similar to the way that biologicals have changed the paradigm of how diseases are treated today [18], gene editing technologies hold great potential for the treatment of a large number of diseases and for the future of drug development. The aim of this article is to give an overview of the current state of clinical development of gene editing-based therapies, as well as to provide an outlook of the gene editing tools that might yet be to come. Furthermore, it discusses the regulatory challenges (and possible solutions thereof) that companies might face during the development of gene editing-based therapies.

### The current gene editing landscape

**Fig. 1:**
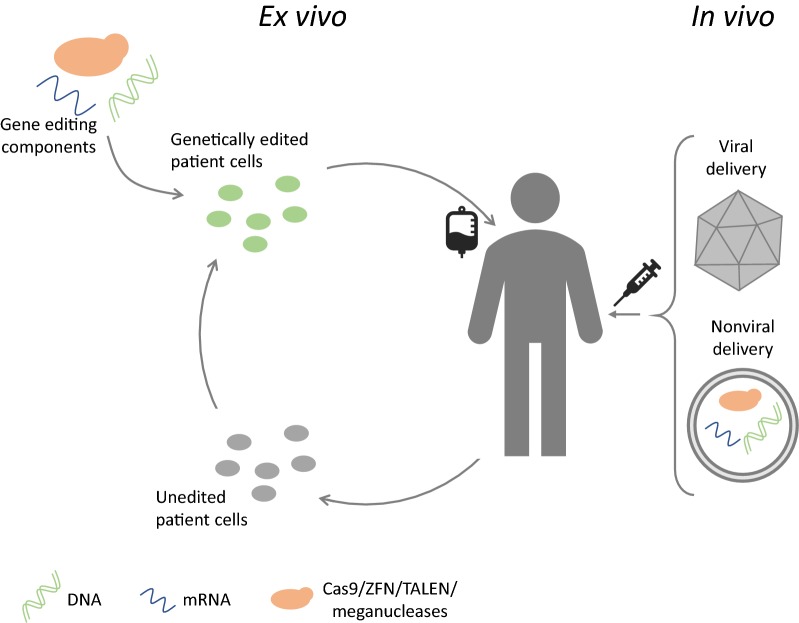
Ex vivo vs. in vivo gene editing. During ex vivo gene editing, the patient’s cells are removed from the body, genetically modified using gene editing components and then transferred back into the patient’s body. Alternatively, e.g. for allogeneic CAR T cell therapies, cells from healthy donors are genetically modified and then transferred into the patient. For in vivo gene editing, gene editing components are delivered directly to the patient’s cells using either viral or nonviral delivery systems

There are a number of ways to describe or classify gene editing therapies. However, most commonly, they are divided into ex vivo and in vivo techniques or processes. During in vivo gene editing, the gene editing components are delivered directly to the cells or organs in the human body, whereas during ex vivo gene editing, the cells are manipulated outside of the body and then transplanted (back) into the patient (Fig. 1).

#### Ex vivo gene editing

Quite a number of clinical trials are currently ongoing around the world that are using the gene editing machinery of ZFNs, TALENs or CRISPR/Cas9 to modify the genome and reach the desired therapeutic result (Table 2). Not surprisingly, the vast majority of these are ex vivo gene editing clinical trials since this approach is the most developed and has advantages over in vivo gene editing in terms of safety and technical feasibility. It is much easier to genetically modify cells that are growing in the laboratory than to deliver the gene editing machinery to a specific subset of cells in the human body. Moreover, there is an additional quality control checkpoint before infusion of the edited cells into the patient, so that doctors have more control over the cell product that they are administering.

**Table 2:** Gene editing clinical trials

	NCT (status)	Country/region	Sponsor	Disease	Target/modification	Nuclease	Delivery
Ex vivo	NCT00842634 (completed)	US	Sangamo Therapeutics	HIV	CCR5 modified CD4+ T cells	ZFN	Adenoviral vector
	NCT01044654 (completed)	US	Sangamo Therapeutics	HIV	CCR5 modified CD4+ T cells	ZFN	Adenoviral vector
	NCT01252641 (completed)	US	Sangamo Therapeutics	HIV	CCR5 modified CD4+ T cells	ZFN	Adenoviral vector
	NCT02388594 (completed)	US	University of Pennsylvania	HIV	CCR5 modified CD34+ T cells	ZFN	mRNA electroporation
	NCT02500849 (active, not recruiting)	US	City of Hope Medical Center	HIV	CCR5 modified CD34+ HSPCs	ZFN	mRNA electroporation
	NCT03617198 (not yet recruiting)	US	University of Pennsylvania	HIV	CCR5 modified, C34-CXCR4, CD4 CAR T cells	ZFN	Not specified
	NCT03666871 (not yet recruiting)	Not specified	Case Western Reserve University	HIV	CCR5 modified CD4+ T cells	ZFN	Not specified
	NCT03190278 (recruiting)	US	Cellectis S.A.	Acute myeloid leukemia	Allogeneic CAR T cells targeting CD123, TCR disruption	TALEN	Not specified
	NCT03164135 (recruiting)	China	Affiliated Hospital to Academy of Military Medical Sciences (China)	HIV-1	CCR5 modified CD34+ HSPCs (from donor)	CRISPR/Cas	Not specified
	NCT03655678 (recruiting)	Canada, Europe	Vertex Pharmaceuticals Incorporated	Beta Thalassemia	Autologous CD34+ HSPCs modified at the enhancer of the BCL11A gene	CRISPR/Cas9	Ribonucleoprotein electroporation
	NCT03728322 (not yet recruiting)	Not specified	Allife Medical Science and Technology Co., Ltd	Beta thalassemia	HBB gene correction in patient specific iHSCs	CRISPR/Cas9	Not specified
	NCT03745287 (recruiting)	US, Europe	Vertex Pharmaceuticals Incorporated	Sickle cell disease	Autologous CD34+ HSPCs modified at the enhancer of the BCL11A gene	CRISPR/Cas9	Ribonucleoprotein electroporation
	NCT03398967 (recruiting)	China	Chinese PLA General Hospital	B cell leukemia, B cell lymphoma	Allogeneic CD19 and CD20/22 CAR T cells	CRISPR/Cas9	Not specified
	NCT03166878 (recruiting)	China	Chinese PLA General Hospital	B cell leukemia, B cell lymphoma	Allogeneic CD19-directed CAR T cells; TCR and B2 M disruption	CRISPR/Cas9	Not specified
	NCT03399448 (recruiting)	US	University of Pennsylvania	Multiple Myeloma, Melanoma, Synovial Sarcoma, Myxoid/Round Cell Liposarcoma	Autologous anti-NY-ESO CAR T cells, disruption of TCR and PD-1	CRISPR/Cas	mRNA electroporation
	NCT03690011 (not yet recruiting)	US	Baylor College of Medicine	T cell Acute Lymphoblastic Leukemia, T cell Acute Lymphoblastic Lymphoma, T-non-Hodgkin Lymphoma	Anti-CD7 CAR T cells, CD7 KO	CRISPR/Cas9	Not specified
	NCT03545815 (recruiting)	China	Chinese PLA General Hospital	Solid tumor	PD-1 and TCR KO anti-mesothelin CAR T cells	CRISPR/Cas9	Not specified
	NCT03747965 (recruiting)	China	Chinese PLA General Hospital	Solid tumor	Mesothelin-directed CAR T cells; PD-1 KO	CRISPR	Not specified
	NCT03081715 (completed)	China	Hangzhou Cancer Hospital	Esophageal cancer	PD-1 KO T cells	CRISPR/Cas9	Not specified
	NCT02793856 (active, not recruiting)	China	Sichuan University	Metastatic non-small cell lung cancer	PD-1 KO T cells	CRISPR/Cas9	Not specified
	NCT03044743 (recruiting)	China	Yang Yang, Nanjing University Medical School	EBV positive advanced stage malignancies	PD-1 KO EBV-CTL cells	CRISPR/Cas9	Not specified
	NCT02863913 (withdrawn—no funding)	China	Peking University	Invasive Bladder Cancer Stage IV	PD-1 KO T cells	CRISPR/Cas9	Not specified
	NCT02867345 (withdrawn—no funding)	China	Peking University	Hormone Refractory Prostate Cancer	PD-1 KO T cells	CRISPR/Cas9	Not specified
	NCT02867332 (withdrawn—no funding)	China	Peking University	Metastatic renal cell carcinoma	PD-1 KO T cells	CRISPR/Cas9	Not specified
In vivo	NCT03041324 (recruiting)	US	Sangamo Therapeutics	Mucopolysaccharidosis II	Insertion of corrected copy of Iduronate 2-Sulfatase gene into the Albumin locus	ZFN	AAV
	NCT02702115 (recruiting)	US	Sangamo Therapeutics	Mucopolysaccharidosis I	Insertion of corrected copy of α-l-iduronidase gene into the Albumin locus	ZFN	AAV
	NCT02695160 (recruiting)	US, Europe	Sangamo Therapeutics	Hemophilia B	Insertion of a corrected copy of the factor 9 gene into the albumin locus	ZFN	AAV
	NCT02800369 (active, not recruiting)	China	Huazhong University of Science and Technology	Human papillomavirus-Related malignant neoplasm	E7	ZFN	Not specified
	NCT03226470 (not yet recruiting)	China	Huazhong University of Science and Technology	Human papillomavirus-related malignant neoplasm	E6, E7	TALEN	Plasmid in gel
	NCT03057912 (unknown)	China	First Affiliated Hospital, Sun Yat-Sen University	Human papillomavirus-Related malignant neoplasm	E6, E7	TALEN CRISPR/Cas9	Plasmid in gel
	NCT03872479 (not yet recruiting)	US	Allergan	Leber congenital amaurosis 10	CEP290	CRISPR/Cas9	AAV

ClinicalTrials.gov was searched for clinical trials involving meganucleases, ZFNs, TALENs or CRISPR/Cas. Current status from July 2019

However, since the cells have to be removed from the patient’s body first, ex vivo gene editing is only suitable for specific cell types. This is also reflected in the current clinical trials, the cell types that are genetically modified and the diseases that they are aiming to cure (Table 2). In all of these ongoing ex vivo trials, the cells that are genetically modified are from the hematopoietic lineages, i.e. either types of T cells or hematopoietic stem or progenitor cells (HSPCs). These cells are not only relatively easy to obtain in sufficient quantity from the patients, they are also readily manipulatable, can be kept in culture without losing their in vivo function and engraft well upon reintroduction into the patient, which makes them ideal candidates for therapeutic gene editing. Typically, these cells are identified and purified using flow cytometry methods. Cells from the hematopoietic lineages are very well characterized in terms of their cell surface markers, which also facilitates their use in gene editing therapies.

##### CAR T cells

One of the biggest success stories of gene therapies so far are likely CAR T cells [19]. These are T cells that are genetically engineered to express a CAR [20, 21] which activates the cytotoxic T cell response upon recognition of cancerous cells. CARs consist of an extracellular antigen-binding domain that can be designed to target any antigen of interest (typically a tumor cell biomarker), a transmembrane domain, as well as an intracellular T cell activating domain (typically CD3ζ with one or two co-stimulatory domains such as CD28, 4-1BB or OX40) [22–25]. The CAR region of the first two approved CAR T therapies, Kymriah^®^ and Yescarta^®^, targets the CD19 antibody which is commonly found in cancerous B cells of leukemia and lymphoma patients. They have been used to successfully treat relapsed/refractory large B cell lymphoma with high remission rates [26–28].

But the new generation of improved CAR T cell products, manufactured using modern gene editing tools, is already on its way—these include allogeneic CAR T cells produced as an “off the shelf” product using healthy donor T cells, as well as CAR T cells with improved functionality.

Allogeneic CAR T cells have a number of advantages over autologous CAR T cells that are produced from the patient’s own T cells, including timely availability, as well as better quality and consistency of the product. As with any cell therapy, however, patient-donor human leukocyte antigen (HLA) mismatch is an issue that researchers needed to tackle to develop successful allogeneic CAR T cells. T cells express receptors on their surface (TCRαβ) that identify any non-self HLA. Therefore, HLA-mismatch between the patient and the donor cells will lead to severe graft versus host and host versus graft responses. One way that scientists are tackling this problem is by using gene editing tools to specifically knockout the *T cell receptor alpha (TRAC)* and *β*-*2 Microglobulin (B2M)* genes in the CAR T cells [29]. The TRAC locus encodes the alpha chain of the TCRαβ while the B2M locus is vital for HLA complex assembly. Indeed, several of the ongoing clinical trials already use this approach (NCT03190278, NCT03166878, Table 2) and their number will most likely increase substantially in the years to come as companies such as CRISPR Therapeutics [30] already have more potential allogeneic CAR T cell therapies coming up in their drug development pipelines.

Looking at the ongoing gene editing (CAR) T cell clinical trials, quite a large number of them also involve knocking out the *Programmed cell death protein 1 (PDCD1)* gene (encodes the PD-1 protein). This is another one of the clever additions to the CAR T cell world, that aims to improve the cells’ functionality by counteracting the immunosuppressive tumor microenvironment and that is owed to the development of gene editing tools. PD-1 is an inhibitory receptor that is expressed on T cells. Its ligands (PD-L1 and PD-L2) are typically expressed on antigen presenting cells, but tumors also often express these ligands and can thereby downregulate the T cell response and avoid the T cell’s tumor-killing activity. Hence, knocking out *PDCD1* from CAR T cells, can eloquently circumvent the tumor cells’ attempt to avoid immune destruction and increase therapeutic efficacy. In fact, the first ever CRISPR/Cas9 clinical trial in China is using T cells (not CAR T cells, however) with a knockout of the *PDCD1* gene (NCT02793856) and others are following (Table 2), including another Chinese study (NCT03747965) that is evaluating the safety and efficacy of mesothelin-directed *PDCD1* knockout CAR T cells for the treatment of solid tumors. Other candidate genes that could be knocked out to reduce downregulation of CAR T cell activity and function by the tumor cells are *CTLA*-*4* [31] and *DGK* [32].

One of the most remarkable gadgets of the gene editing toolbox is that CRISPR/Cas9 allows for simple multiplexing of gene editing [9, 29]. In the future we will probably see many more clinical trials with CAR T cells that were extensively edited including multiple gene knockouts and/or knockins, aiming at creating safer and more efficacious gene therapy products.

##### Gene editing in hematopoietic stem and progenitor cells

For the same reason that T cells are a popular tool for the development of therapies using gene editing, HSPCs are also gaining popularity in the field of gene therapies. Several ongoing clinical trials show that there is a lot of potential for using genetically edited HSPCs for the treatment of diseases of the hematopoietic system.

The first ever clinical trial using gene editing technologies was completed in 2014 (NCT00842634) [33] and was investigating the use of autologous CD4+ T cells with a ZFN-mediated knockout of the *co*-*receptor chemokine 5 (CCR5)* gene for the treatment of HIV. CCR5 is a chemokine that is expressed on the cell surface of lymphocytes and serves as one of the main co-receptors of the CD4 receptor that allows entry of HIV into the cell [34–38]. It had been shown previously that people with a mutation in the *CCR5* gene are resistant to HIV infection [39–41] and therefore, this was an attractive candidate gene to target using gene editing strategies. After preclinical studies in mice confirmed the use of *CCR5* knockout CD4+ T cells for the treatment of HIV, a clinical trial was also initiated in humans. This study confirmed that this treatment is generally safe. However, due to low gene editing efficiencies and subsequently low numbers of *CCR5* knockout T cells, the therapeutic effect was disappointing and therefore, more studies are currently ongoing to optimize the treatment (NCT01044654, NCT01252641, NCT01543152, NCT02225665, NCT02388594). One approach to optimize this treatment method was the choice to use *CCR5* knockout HSPCs instead of CD4+ T cells (NCT02500849). Since HSPCs have the feature that they can renew as well as differentiate into all cells of the hematopoietic lineages, this has two advantages over using T cells for HIV treatment. Firstly, although CD4+ T cells are the main access point for HIV, other HIV-susceptible cells such as CD4+ myeloid cells can also be protected from HIV infection. Secondly, this would potentially produce an unlimited source of *CCR5*-knockout T cells, improving the long-term efficacy of such a treatment. HIV (or the disease that it causes, AIDS) has been a challenge for clinicians and researchers alike for many years and it continues to be so. Although antiretroviral therapies are able to keep the virus in check, no curative therapy is approved so far. However, using gene editing tools to produce a knockout of a single gene in HSPCs may bring us closer to the solution.

Two other diseases that have received a lot of attention recently are sickle cell disease (SCD) and β-thalassemia, as a drug developed by CRISPR Therapeutics and Vertex Pharmaceuticals is the first CRISPR/Cas9 drug to go into clinical trials in the US and Europe (Table 2, NCT03655678, NCT03745287). These are monogenic, inherited diseases that are caused by mutations in the *β*-*globin* gene [42, 43], resulting in faulty synthesis or mutant variants of the β-globin chain of hemoglobin. Patients suffer from severe and often life threatening symptoms including anemia. Although the genetic etiology as well as the clinical course of disease of both SCD and β-thalassemia is well understood, developing treatments for these conditions has been challenging with the only currently available therapy being allogeneic HLA-matched hematopoietic stem cell transplants. Unfortunately, HLA-matched donors are unavailable for most patients and hence these diseases still remain a burden for both patients and doctors. However, this may change soon thanks to genetically edited HSPCs. Interestingly, it has been shown that a fetal globin (γ-globin) can functionally replace β-globin in SCD and β-thalassemia [44–47]. Expression of γ-globin is downregulated after birth and replaced with the adult version β-globin. This switch is mediated through the activation of the transcriptional regulator BCL11A, a suppressor of *γ*-*globin* gene expression [48]. Hence, knockout of *BCL11A* in the patients’ HSPCs has been identified to be a suitable gene editing candidate for the treatment of the hemoglobinopathies SCD and β-thalassemia as it may provide a method to provide effective long-term treatment for a large number of patients. Both CRISPR/Cas9 studies, as well as an additional ZFN study by Sangamo (NCT03432364) use gene editing tools to mutate an erythroid enhancer of *BCL11A* in patient HSPCs. This clever strategy specifically knocks out *BCL11A* expression in erythroid cells (hemoglobin producing cells) and ensures that *γ*-*globin* is reactivated in the erythroid lineage, while protecting functionality of non-erythroid cells which have been shown to require *BCL11A* expression. Such manipulation of the genome would not have been possible with traditional gene therapy. The first patients are being treated under these clinical trials and it will be very exciting to see the outcome of those studies as they may well become precedent cases for the treatment of monogenic diseases using genetically edited HSPCs.

Other genetic diseases that could potentially be treated by employing this technology include the hundreds of genetic disorders of the hematopoietic and immune systems. A lot of research is ongoing for X-linked severe combined immunodeficiency (SCID-X1; mutation in the *IL*-*2 receptor common gamma*-*chain* [*IL2RG*] gene) [49–51] and X-linked chronic granulomatous disease (X-CGD; mutation in the phagocyte nicotinamide adenine dinucleotide phosphate oxidase [NADPH] complex subunits) [52] and it can be expected that several clinical trials will be initiated in those indications in the next couple of years.

In the future, once molecular markers of additional tissue specific stem cells are known and cell extraction and culture techniques have advanced to a stage that enable us to grow these cells, gene editing technologies could also be used to modify those tissue stem cells and potentially produce stem cell based ex vivo gene therapies for a greater variety of diseases.

##### Gene editing as an antiviral treatment

HIV is also the target indication of the work of Hauber and Buchholz, who are using another gene editing tool, called engineered tyrosine recombinase, to specifically eliminate the HIV provirus from infected CD4+ T cells [53]. They have used directed evolution to produce the broad-range recombinase 1 (Brec1) that recognizes a specific 34 bp sequence located within the long terminal repeats of HIV and is therefore able to efficiently excise the HIV provirus. This has been successfully demonstrated in preclinical in vitro and in vivo models of HIV. This potential therapy is still in early stages, but it is another excellent example of the possibilities of gene editing tools. It shows that they can not only be used to treat diseases caused by mutations in the patient’s own DNA, but that they can potentially also be deployed as antiviral therapies.

#### In vivo gene editing

The vast majority of disease are not treatable using ex vivo genome editing. For those diseases, scientists are doing their utmost to develop in vivo gene editing strategies that deliver the gene editing machinery directly to the target cells (Fig. 1). Although ex vivo gene editing techniques are more advanced, in vivo gene editing still offers enormous potential once the technical challenges that currently limit its use are tackled.

##### In vivo gene editing in practice

In fact, recent advancements and improvements of in vivo gene editing methods have enabled development of the first in vivo gene editing drugs that are being investigated in clinical trials now. In November 2018, the first patient was treated as part of Sangamo’s clinical trial in patients with Mucopolysaccharidosis II (NCT03041324). Mucopolysaccharidosis II is caused by mutations in the *deficiency of iduronate*-*2*-*sulfatase (IDS)* gene and can lead to life threatening tissue and organ damage. The drug developed by Sangamo is a ZFN that inserts a copy of the *IDS* gene into the *Albumin* locus with the aim of restoring IDS enzyme activity. They use an AAV vector that specifically targets liver cells, from where the corrected protein is released into the blood stream. This is supported with data from their preclinical studies [54]. Preliminary results from their clinical study show evidence of successful in vivo gene editing with increased levels of IDS enzyme in patients. Two similar ZFN products developed by Sangamo are also currently in clinical trials for the treatment of Mucopolysaccharidosis I (NCT02702115) and Hemophilia B (NCT02695160).

Even more recently, the first in vivo CRISPR/Cas9 gene editing trial was announced by Allergan for the treatment of Leber Congenital Amaurosis (LCA) 10 (NCT03872479). LCA 10 is a retinal degenerative disease caused by mutations in the *CEP290* gene that results in childhood blindness. The most common mutation, which is also being targeted in this clinical trial, is a point mutation that creates an aberrant splice site and subsequently results in abnormal assembly of photoreceptors [55–57]. Using subretinal injection of an AAV5 vector (which shows tropism for cells of the retina, including photoreceptor cells) CRISPR/Cas9 gene editing machinery is delivered to the target cells. Using those tools, DSBs are made at either side of the novel splice site and the sequence will be removed or inverted, which is expected to restore normal CEP290 activity. This has been shown to be effective in a mouse model of the specific mutation [58].

It will be extremely interesting to see what the outcome of these clinical trials will be as they may well be used as precedent cases for future in vivo gene editing therapies. There are many, potentially hundreds if not thousands of other diseases, that may be cured in the future using in vivo gene editing technologies and for many, preclinical studies are already ongoing.

One disease that is receiving a lot of attention as a potential target for in vivo gene editing is Duchenne muscular dystrophy (DMD). DMD, a severe neuromuscular disorder, is one of the most common lethal genetic disorders in humans, yet to this day there is no cure for this disease [59]. Patients typically present with muscle weakness during early childhood which progresses to muscle degeneration including symptoms such as loss of motor skills and ability to walk, problems with breathing and eventually death. Some patients also present with neurobehavioural disorders and cognitive impairment. DMD is caused by mutations in the *dystrophin* gene. Dystrophin links the cytoskeleton of the muscle cell to its extracellular matrix and acts as a “damper” to protect muscle fibers during muscle contractions. In its absence, muscle fibers are damaged [60, 61]. The *dystrophin* gene is the largest known human gene spanning approximately 2.3 megabases [62] and consisting of 79 exons [63]. To date, more than 3000 mutations are known, including insertions, deletions, duplications and point mutations [64–66]. This combination makes the development of gene editing therapies for the treatment of DMD extremely difficult. Interestingly, the vast majority of these mutations cluster around exons 45–55, the so called hotspot region [67]. Most mutations are out of frame mutations that produce premature stop codons, resulting in a truncated protein and the severe symptoms of DMD. Occasionally, mutations cause internal in frame mutations. These cause a much milder form of DMD—Becker muscular dystrophy [68]. Most therapeutic approaches of in vivo genome editing of the *dystrophin* gene aim to mimic this milder phenotype and rely on exon-skipping to restore the reading frame. This can be done by using gene editing tools to produce DSBs on either side of the region that should be deleted. Non-homologous end joining will then join the two ends back together, to produce an in frame functional protein. It has been suggested that skipping exons 45–55 could potentially provide a treatment option for 62% of DMD patients [69]. Several preclinical mouse studies have provided proof-of-concept for this approach to treat DMD [70–74] and companies such as CRISPR Therapeutics and Vertex Pharmaceuticals are also already looking into in vivo gene editing therapies for DMD [75]. It can only be a matter of time until these efforts are translated into the clinic.

### Improving the toolbox—the next generation of gene editing

**Fig. 2:**
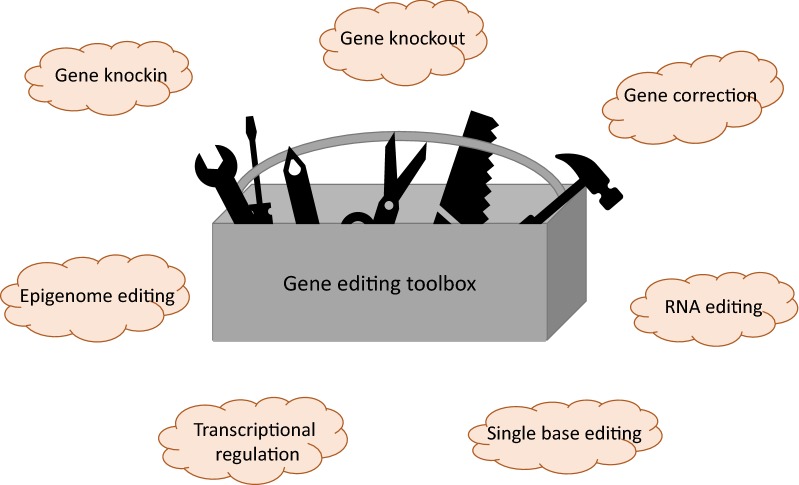
Gene editing toolbox

Although current gene editing technologies that are being used in and developed for clinical applications are offering potential treatment strategies for a vast amount of diseases, the next generation of gene editing tools is already on its way. Figure 2 provides an overview of some of the tools offered by gene editing technologies.

#### CRISPR/Cas for epigenome editing and transcriptional regulation

For a long time, it was thought that misregulation of gene expression is solely due to changes in the DNA sequence itself. However, it is now clear that this does not show the whole picture and that other modifications such as DNA methylation or histone modifications (to only name a few), collectively known as epigenetics, also play an important role in gene expression regulation.

Traditionally, gene editing technologies have only been used to modify the genome. However, in recent years more and more tools are being developed to also modify the epigenome. The basis for this work was the “nuclease-dead” Cas9 (dCas9) which had been engineered to be inactive by introducing mutations into both of its catalytic domains (RuvC and HND) [1, 76]. dCas9 still has the capacity to be targeted to and bind to a specific DNA sequence, but through the loss of its catalytic function, it is unable to introduce DSBs. This inactive dCas9 has been utilized by a number of research groups to develop fusion proteins of dCas9 and epigenetic effectors [77–82]. With the help of dCas9, these can be directly targeted to the locus of interest and hence this system enables targeted editing of the epigenome.

Epigenetic effectors can be classified into those that modify histone modifications and those that have an effect on DNA methylation or demethylation. For example, Liu et al. [82] have demonstrated that fusing the de novo DNA methyltransferase 3a (Dnmt3a) or the methylcytosine dioxygenase Tet1 to dCas9 enables targeted methylation or demethylation and subsequent functional repression or activation of genes, respectively. Similarly, Hilton et al. [78] engineered a dCas9 fusion with the histone acetyltransferase P300, which induced H3K27ac (a marker associated with active transcription) with high specificity and robustly activated transcription of the targeted genes.

Epigenetic biomarkers are attracting increasing interest from researchers and clinicians and indeed, a large number of conditions have been implicated with alterations in the epigenome. These include *BRCA1* methylation in breast and ovarian cancer, *RARB2* methylation in lung cancer or histone modifications in Huntington’s disease to name just a few. Moosavi and Motevalizadeh Ardekani [83] and Berdaso and Esteller [84] provide a detailed overview of diseases caused by an abnormal epigenome. Several epigenetics-based therapies are currently under investigation and some DNMT inhibitors (DNMTi) and histone deacetylase inhibitors (HDACi) are approved in the US and EU for the treatment of oncological indications. For example, in 2004 the DNMTi Vidaza^®^ became the first epigenetics-based therapy to be approved in the US. Its active substance is azacytidine, which acts as a broad DNA demethylating agent leading not only to reactivation of the disease-causing genes, but also to unwanted activation or modifications of other sequences. This lack of specificity is a common problem with epigenetics-based therapies and they are typically accommodated by a long list of unpleasant and dangerous side effects [85].

Using CRISPR/dCas9 based epigenome editing techniques this issue could potentially be circumvented as they provide a way to target epigenetic effectors directly to the site(s) of interest. These methods will likely find applications in a wide range of diseases including cancers, neurodevelopmental and neurodegenerative diseases as well as imprinting disorders. Some of these are receiving a lot of attention, as they could not yet be cured by alternative therapies. The same is true for a lot of complex diseases, that are being unraveled slowly using genome wide association studies (GWAS). The majority of variants that are detected by GWAS are located in non-coding regions of the genome and are enriched with epigenomic marks. The role of these regions is still unclear for most disorders. However, using epigenome editing techniques, they could eventually become interesting targets for drug development.

In the same way that dCas9 can be employed to target epigenetic effectors to specific genomic loci, gene editing tools can also be used to precisely deliver transcriptional modulators to promoter regions of genes such as the transcriptional repressor Kruppel-associated box (KRAB) or the transcriptional activator domain VPR. In this way, gene expression can be selectively activated or repressed [76, 86]. Proof-of-concept that such repurposing of gene editing tools can be used to change the transcriptome and ameliorate disease status, has been shown in a number of animal models of disease [87–90].

#### Base editing

The catalytically inactive dCas9 has not only been used in the context of epigenetics and transcriptional regulation, but it has also been developed to edit single bases without having to induce DSBs. Researchers have engineered fusion proteins consisting of dCas9 with cytidine deaminase [91, 92] and dCas9 with tRNA adenine deaminase [93] which convert C to T and A to G, respectively. Advantages over standard CRISPR/Cas9 techniques include higher correction efficiencies as well as fewer off-target effects [91]. Although they are not used in the clinical setting yet, these methods could be particularly useful for the treatment of point mutations, which are often the cause of monogenic diseases.

#### RNA editing

While Cas9 is a DNA-specific nuclease, the related Cas13 nuclease can be used to enable alterations of the RNA [94]. The function of Cas13 is similar to that of Cas9. However, unlike Cas9, which induces cleavage of the DNA, the Cas13 family cleaves RNA. One major concern with all gene editing technologies are deleterious or potentially lethal off-target effects. In contrast, RNA editing with Cas13 does not induce permanent changes in the DNA, while still having an effect on protein function or abundance.

Although this technique is certainly an excellence tool for research, it is still unclear what the potential is for the clinical setting. Due to the RNA’s relatively short half-life, these RNA editing tools would need to be administered repeatedly to maintain a therapeutic result.

## Challenges and solutions

Overall, it has been shown by both the current ongoing clinical trials as well as the preclinical work that is going on in research institutes around the world, that gene editing has tremendous potential to provide a cure for a vast number of diseases for which we are still lacking therapies to date. However, as with any new, emerging technology, translating it to the clinic is associated with some challenges that need to be addressed. These include technical challenges, such as off-target effects and the improvement of delivery systems for in vivo gene editing, as well as regulatory considerations.

### Technical challenges

Two of the main technical challenges that still remain with gene editing technologies are: firstly safe and efficient delivery of the editing components to the target cells and the need for spatiotemporal control over the expression of the nucleases, and secondly the specificity and precision of the gene editing machinery.

#### Delivery systems for in vivo gene editing

While delivery of the gene editing components is not such a big problem for ex vivo gene editing, it is one of the biggest obstacles for the development of in vivo gene editing therapies and a lot of research is going on to improve the delivery systems. Biagioni et al. provide a detailed review of the current state of such delivery systems for gene editing technologies [95]. The systems that are being developed can be divided into viral and nonviral delivery systems. Viral systems include lentiviruses, adenoviruses and AAVs. Nonviral delivery systems are mainly nanoparticles such as liposomes (Table 3).

**Table 3:** Comparison of gene editing delivery systems

Vector type	Advantages	Disadvantages	Solution/developments
AAV	Good tissue tropism Efficient gene editing Low immunogenicity	Low packaging capacity	Trans-splicing vectors
Adenovirus	Large packaging capacity	High immunogenicity Low tissue tropism	Removal of viral genes to avoid immunogenicity
Lentivirus	Large packaging capacity	Stable integration into genome Low tissue tropism	Non-integrative lentiviral vectors Can be engineered to show tissue tropism
Nonviral delivery systems (e.g. lipid-based nanoparticles)	Delivery of DNA, rRNA or protein complexes and transient expression Virus free	Naturally low tissue tropism Limited delivery efficiency	Development of systems with high tissue tropism Improvement of efficiency

So far, AAV has been the vector of choice for delivery of the gene editing machinery to the target cells (Table 2). This is due to several reasons: most importantly, AAV exists in a number of structurally different serotypes with distinct tissue tropisms, allowing for gene editing in specific tissues and cell types [96]. Chemical and genetic modifications of AAV capsids are also being used to further increase the specificity towards certain cell types [97–99]. Additionally, AAV is very efficient and its DNA does not typically integrate into the host genome, which makes it a relatively safe delivery vector and limits the chance for insertional mutagenesis. Finally, compared to other viral vectors, AAV induces only a very low immune response in the patient. However, the main disadvantage of AAV as a delivery vector is its low packaging capacity of only up to 5 kb. This limits the packaging of large nucleases such as TALENs or some Cas9 nucleases, as well as the delivery of both a nuclease and a donor DNA template for gene knockin/correction by homology-directed repair. To solve this, so called trans-splicing vectors have been designed that recombine within the target cells and allow for the delivery of larger DNA sequences. Unfortunately, so far the efficiency is considerably lower than using a single AAV vector [100]. ZFNs are much smaller than TALENs and Cas9 nucleases which facilitates their packaging into AAV vectors.

Adenovirus and lentivirus vectors both have the advantage that they have a much larger packaging capacity and can deliver all parts of the nuclease (plus sgRNA in the case of CRISPR/Cas9), as well as a donor DNA template. Therefore, all parts of the gene editing machinery are going to be expressed in the cells together, which tremendously increases efficiency compared to delivery by several AAVs. Another strong advantage of lentiviruses is that like AAV vectors they can be engineered to show tropism to cell types of interest. Traditionally, these vectors do not come without drawbacks, but developments in recent years are creating viral vectors that are greatly improved. While delivery by adenoviruses leads to transient expression of the nuclease and donor template, lentiviral vectors often stably integrate into the genome. This can be acceptable for traditional gene therapy which only delivers an exogenous donor template, but continuous expression of nucleases can be a serious safety concern due to off-target effects, in particular in the presence of a donor template. In recent years, non-integrative lentivirus vectors have been developed [101, 102], making lentiviruses more attractive for gene editing. The major drawback of adenoviruses is that they can often cause severe immune responses in patients, but it was shown that removal of the viral genes can bypass this effect, making it much more useful for the application in in vivo gene editing therapies [103, 104].

Nonviral systems for the delivery of gene editing machinery are also being developed. For ex vivo gene editing, electroporation of mRNA, nucleases and also Cas9/sgRNA ribonucleoprotein (RNP) complexes is commonly chosen (Table 2), but the feasibility is limited for in vivo gene editing. Other nonviral delivery systems include lipid- or polymer-based nanoparticles such as liposomes that can be used to package gene editing cargo. The advantage of such methods over viral delivery is that they can not only deliver DNA to the target cells but also mRNA and protein complexes, such as the Cas9/sgRNA ribonucleoproteins [105, 106]. These will only be transiently expressed, greatly limiting safety concerns compared to viral delivery of the nucleases. Although such delivery systems are not used in clinical trials yet, a lot of research is looking into improving efficiency and finding ways to target them to specific tissues. Indeed, systems have already been developed that show high efficiency and tissue specificity, such as the cKK-E12 lipopeptide which has been used for siRNA delivery to the liver in rodents and nonhuman primates [107] as well as more recently for the delivery of Cas9 mRNA and sgRNA to the liver, resulting in highly efficient editing of the target gene [108].

More research and development is surely needed, but both optimized viral and nonviral delivery systems could change the field of gene editing therapies by providing safer and more efficient delivery methods for in vivo as well as ex vivo gene editing.

#### Specificity—potential for off-target effects

One of the biggest concerns with genome editing tools are off-target effects and their consequences. Although nucleases can be designed to induce DSBs at the target site with high specificity, there is a residual risk for off-target mutations, i.e. insertions or deletions away from the intended locus. Off target mutations can occur if the nucleases (or sgRNA in the case of CRISPR) recognize sequences that are similar to the target sequence. Like with any random insertion or deletion of genetic material, this can give rise to deleterious, often carcinogenic mutations.

However, in the same way as the gene editing toolbox is constantly being improved to allow for modifications of more diverse targets (such as epigenome editing), a lot of research, especially in the field of CRISPR, also focuses on enhancing accuracy and minimizing off-target effects of gene editing tools. These new variants of the CRISPR/Cas9 technology include inducible CRISPR/Cas9 systems [109–111], inhibition of the CRISPR/Cas9 system using anti-CRISPR [112] and engineered Cas9 variants such as Cas9 nickase (nCas9) [113, 114], Fok1-dCas9 fusions [115, 116] or split-Cas9 [117, 118]. All these systems have been shown to have improved gene editing specificity and reduced off-target activity.

Nevertheless, companies still need to seriously consider the risk for off-target effects during their lead candidate selection process and preclinical development. As development of gene editing technologies progresses, so do the tools to assess and predict the specificity of gene editing at the intended site (on-target) as well as potential off-target editing. They include extremely sensitive methods based on high-throughput sequencing combined with state of the art bioinformatics tools. The review by Tsai and Joung [119] provides an overview of some tools and methods that are available to date. However, not only the potential off-target events need to be identified but their likely effect on the functionality of the cells and subsequently their clinical implications also need to be assessed and validated during the preclinical stage of drug development.

### Regulatory

Gene editing technologies as a therapeutic product class are relatively new. Therefore, companies often raise the question how such products should be regulated. In the EU, gene edited products fall under the regulatory framework of advanced therapy medicinal products (ATMPs) which has been in place since 2008 (Regulation (EC) No. 1394/2007).

Compared with other product classes, ATMPs had a relatively bad start into the regulatory world with a disappointing success rate of only 60% at the time of authorization which is considerably lower than other product classes (80 to 90%). Between 2009 and March 2019, 14 products gained market authorization, but four of these products have already been withdrawn, mainly due to commercial reasons, so that only 10 ATMP products are currently available [120]. Considering that there are hundreds of ongoing trials using ATMPs, this number seems incredibly low. But why is that and what can be improved?

The European Medicines Agency (EMA) has published a number of guidance documents and reflection papers on ATMPs that can be of great help to companies developing gene editing products. These include guidance on requirements for product quality, as well as preclinical and clinical development. Cathomen et al. [121] also provide an overview of what regulators expect in genome editing clinical trials. However, Agencies still observe that for ATMPs there often are issues in all parts of the product dossier. For the quality development, they include inconsistent manufacture, deficiencies in the product characterization and potency assays, and insufficient comparability following manufacturing changes. One of the major shortcomings during nonclinical development is the lack of suitable animal models to demonstrate safety, biodistribution and pharmacodynamic properties of the therapeutic agent. Alternative strategies identified are often not accepted to be suitable by the Agencies. In terms of clinical development, the main issues are usually lack of valid therapeutic endpoints, the selection of comparator or the lack of proper statistical analysis (often due to limited patient numbers).

However, by using strategic upfront planning and considering these hurdles early on during product development, it is possible to turn what seems like regulatory challenges into important considerations that should be addressed during drug development (Table 4). Examples are smart selection of the lead and back-up candidate in view of the on-target/off-target profile, an integrated on-target/off-target assessment report as part of the drug screening and evaluation approach and as a basis for clinical trial applications, a comprehensive benefit/risk assessment including mitigation strategy, a tailored nonclinical program, and a well thought out manufacturing and control strategy for the drug product as well as critical materials, such as editing materials. Furthermore, a diligent plan towards the first in human study including risk mitigation strategy and involvement of regulatory Agencies at defined milestones should be considered as well to increase the success rate. The set-up of an integrated development and regulatory plan is a valuable approach towards structured drug development and discussions in a multidisciplinary team ensure that no essential element is missed.

**Table 4:** Important considerations during gene editing-based drug development

Product dossier section	Common challenges	Solutions
Quality	Inconsistent manufacture Insufficient product characterization Deficiencies in potency assays Lack of comparability after manufacturing changes	Strategic manufacturing and control strategy for the drug product as well as critical materials Integrated development and regulatory plan Consider related benchmark cases
Nonclinical	Lack of sensitive and relevant models Difficulty to assess safety and pharmacodynamic properties Unsuitable alternative strategies and justification thereof	Smart selection of the lead and back-up candidate Tailored nonclinical program Integrated on-target/off-target assessment report
Clinical	Lack of valid therapeutic endpoints Selection of comparator Lack of proper statistical analysis	Comprehensive benefit/risk assessment Diligent plan towards the first in human study Involvement of regulatory Agencies

In practice, it is always advisable to consider related benchmark cases. Companies developing gene editing therapies will face similar, if not the same, challenges as with ATMPs and although gene editing therapies have not been approved yet so that precedent cases are limited, we can still learn a lot from the experience with ATMPs. For example, when developing a CAR T therapy using genome editing, experience from other approved CAR T cell therapies such as Kymriah^®^ or Yescarta^®^ combined with published experience from gene editing products should be considered.

## Conclusion

The great clinical potential of gene editing technologies has led to an increase in development of novel and improved therapies for diseases that so far lack effective interventions. The advantages of gene editing-based therapies over traditional gene therapy are the precise targeting of specific loci, as well as the more variable toolkit which does not only allow for the random insertion of exogenous DNA but also enables precise gene knockouts, gene replacements, targeted insertion of genetic material, single base editing as well as targeted changes to the epigenome. Although gene editing-based therapies are not approved yet, a number of them are being explored in clinical trials and their number is steadily increasing. However, technical and regulatory hurdles remain and it is recommended for companies to consider these challenges early on during drug development in order to avoid deficiencies in the product dossier and have a successful marketing authorization application. We highly recommend setting up smartly integrated drug development plans, diligent on-target -/off-target assessment strategies as well as a well thought out risk mitigation plan.

## Data Availability

Not applicable.

## Abbreviations

AAV: adeno-associated virus
ATMP: advanced therapy medicinal product
B2M: β-2 microglobulin
CAR: chimeric antigen receptor
Cas: CRISPR-associated nuclease
CRISPR: clustered regularly interspaced short palindromic repeat
DMD: Duchenne muscular dystrophy
DNMT: DNA methyltransferase
DNMTi: DNMT inhibitor
DSB: double strand break
EMA: European Medicines Agency
FDA: US Food and Drug Administraiton
GWAS: genome wide association study
HDACi: histone deacetylase inhibitors
HLA: human leukocyte antigen
IDS: iduronate-2-sulfatase
KRAB: Kruppel-associated box
RNP: ribonucleoprotein
SCD: sickle cell disease
TALEN: transcription activator-like effector nuclease
TCR: T cell receptor
TRAC: T cell receptor alpha
ZFN: zinc finger nuclease
